# Ebola Virus Persistence in Semen Ex Vivo

**DOI:** 10.3201/eid2202.151278

**Published:** 2016-02

**Authors:** Robert J. Fischer, Seth Judson, Kerri Miazgowicz, Trent Bushmaker, Vincent J. Munster

**Affiliations:** National Institutes of Health, Hamilton, Montana, USA

**Keywords:** Ebola virus disease, semen, sexual transmission, qRT-PCR, Ebola diagnostics, viruses, Ebola

## Abstract

On March 20, 2015, a case of Ebola virus disease was identified in Liberia that most likely was transmitted through sexual contact. We assessed the efficiency of detecting Ebola virus in semen samples by molecular diagnostics and the stability of Ebola virus in ex vivo semen under simulated tropical conditions.

On March 20, 2015, an isolated Ebola virus disease (EVD) case was diagnosed in Liberia, 30 days after confirmation of the previous EVD case (the incubation period for Ebola virus [EBOV] infection is 4–21 days). The patient had no history of travel to areas with reported EVD, no interaction with visitors from Sierra Leone or Guinea, no funeral attendance, and no contact with a patient with EVD symptoms (*1*).

The patient, a 44-year-old woman, reportedly had unprotected sex with a male survivor of EVD (*1*). His semen was positive for EBOV by real-time quantitative reverse transcription PCR (qRT-PCR) 199 days after symptom onset; his cycle threshold (C_t_) value was 32, seven days after EBOV was confirmed in the woman (*1*).

Among the criteria for declaring an end to the Ebola outbreak in West Africa, the World Health Organization includes testing of semen of convalescent men until 2 samples are negative (*2*). Most of these specimens will be analyzed by qRT-PCR. Therefore, during May–September 2015, we analyzed the stability of EBOV in semen by qRT-PCR and titration. Because most of the EVD diagnostic laboratories are more familiar with values obtained from blood, we compared standard curves of C_t_ values with the 50% tissue culture infectious dose (TCID_50_) per mL in semen, blood, and tissue culture medium.

## The Study

Human semen and blood were obtained from Lee Biosolutions (St. Louis, MO, USA). All assays were consistent with the procedures used at the Centers for Disease Control and Prevention/National Institutes of Health laboratory at the Eternal Love Winning Africa campus in Monrovia, Liberia, to diagnose EVD in the 44-year-old woman. RNA was extracted by using a QIAamp Viral RNA Mini Kit (QIAGEN, Valencia, CA, USA) following the manufacturer’s protocol, with an additional wash step of wash buffer 1. qRT-PCR was conducted by using Roche LightCycler 480 RNA Master Hydrolysis Probes (Roche, Indianapolis IN, USA) reagents with primers and probes targeting the L gene of EBOV on the SmartCycler (Cepheid, Sunnyvale, CA, USA) platform (*3*).

**Figure 1:**
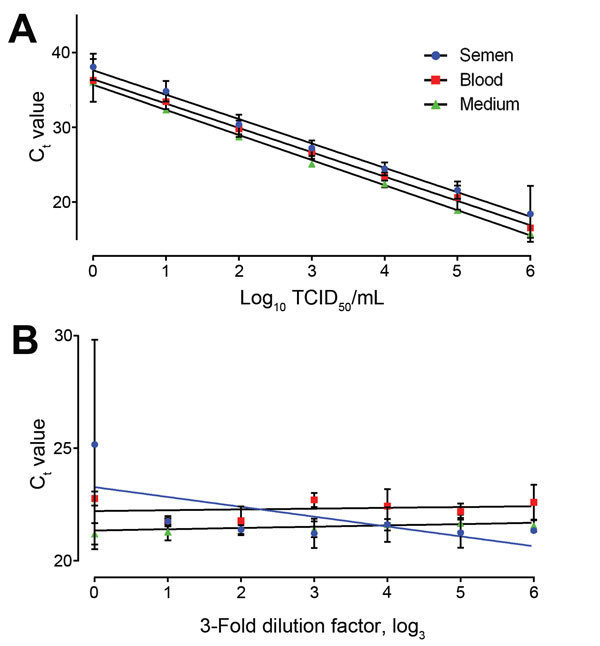
A) Standard curves of Ebola virus spiked into 3 matrices: semen, blood, and tissue culture medium. Samples were analyzed on the basis of 5 biologic replicates. PCR efficiency was from 98% in cell culture medium, 102% in semen, and 103% in blood. Analysis of covariance showed no significant difference (p<0.05) between the slopes of the linear regressions of blood and semen. B) Matrix dilution in which semen, blood, and tissue culture medium were 3-fold serially diluted in sterile physiologic saline solution and spiked with 10^4^ TCID_50_/mL Ebola virus. The slopes for blood and medium did not differ significantly from 0. If the semen sample data are analyzed disregarding the undiluted sample, the resulting slope also does not differ significantly from 0. C_t_, cycle threshold; TCID_50_, 50% tissue culture infectious dose. Error bars represent the mean ± SEM of 5 quantitative PCR analyses.

Ebola virus/H.sapiens-tc/GUI/2014/Makona-WPGC07 was used in all experiments. To enable comparison among the C_t_ values of EBOV in semen with samples routinely analyzed during the current outbreak, we constructed standard curves of EBOV in semen, blood, and medium. Matrices were spiked to 10^6^ TCID_50_/mL, then serially diluted 10 times. Five biologic replicates were used to construct the curves (Figure 1, panel A). The dynamic range of the assay extends down to 10° for blood and semen. The PCR efficiency determined from the slope of the standard curve was nearly 100% for each of the matrices; the C_t_ value was 1.2 times higher on average for semen than for blood.

We tested the stability of EBOV in human semen in the liquid (bulk) and dry states during an 8-day period (27°C, 80% relative humidity [RH]). EBOV was diluted in triplicate in semen to 1 × 10^6^ and 1 × 10^3^ TCID_50_/mL; 50-μL aliquots of semen were removed daily and placed into 450 μL of DMEM. Additional aliquots were obtained for qRT-PCR. To assess the stability in dried semen, 50 μL of spiked semen was spread onto the bottom of each well of a 24-well plate and recovered by resuspending in 500 μL of DMEM. To assess the viability of EBOV in condoms, 700 μL of semen spiked with 1 × 10^3^ TCID_50_/mL EBOV was placed in condoms (Durex Extra Sensitive; Reckitt Benckiser Group, Slough, UK) in triplicate, stored at 27°C and 80% RH, and sampled on alternate days. All samples were stored at −80°C until titration. We previously had determined no significant effect on EBOV titers by a single freeze/thaw step (*4*).

Titrations were performed on Vero E6 cells in a 48-well format. TCID_50_ per milliliter was calculated by using the Spearman-Karber method (*4*,*5*). Statistical analysis were performed with GraphPad 6.05 (GraphPad Software, San Diego, CA, USA).

Standard curves for EBOV in semen and blood did not significantly differ (analysis of covariance, p = 0.8965) between the slopes of the standard, indicating that the PCR efficiency is similar between the 3 matrices; however, differences in C_t_ value between semen and blood were significant (analysis of covariance, p<0.0001) (Figure 1, panel A) (*6*). No linear correlation was observed between the C_t_ value and the dilution factor, together with the additional wash step used during the extraction procedure; this finding suggests that the differences were not due to presence of inhibitors but rather to the efficiency of extraction (Figure 1, panel B). The decrease in extraction efficiency could be related to the nonhomogeneous nature of semen. SE within undiluted samples was larger in semen than in blood (Figure 1, panel B). This finding was confirmed by similar SE variation for the housekeeping gene B2M (semen C_t_ value 28.34 ± 3.35 and blood C_t_ value 20.78 ± 0.81).

**Figure 2:**
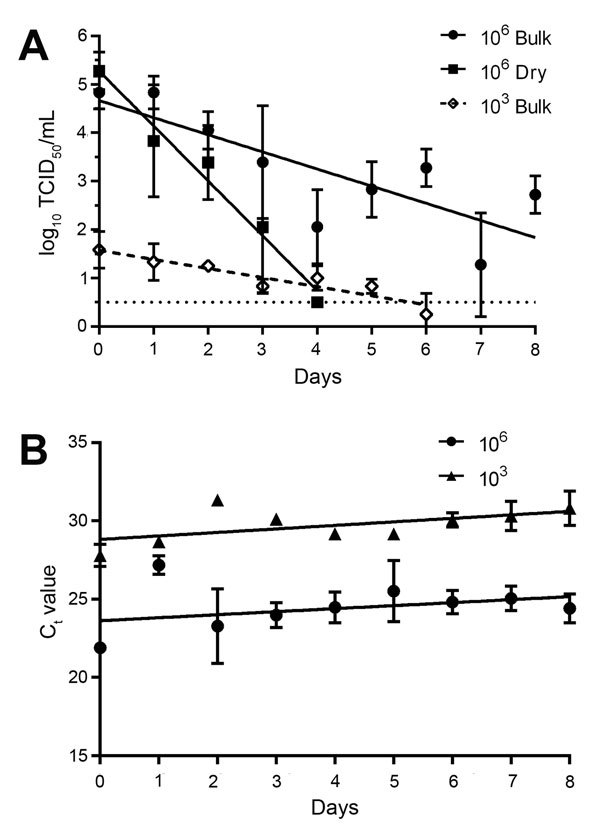
Linear regression model showing stability of Ebola virus (EBOV) and EBOV RNA in semen at 27°C and 80% relative humidity over 8 days. A) EBOV in bulk (liquid) semen versus dry semen at initial titers of 10^6^ TCID_50_/mL and 10^3^ TCID_50_/mL. The higher titer 1 × 10^6^ TCID_50_/mL was used to provide a comparison with EBOV in blood, and the lower titer 1 × 10^3^ TCID_50_/mL was derived from C_t_ values reported in semen samples. Viable virus was reduced significantly faster (p<0.0001) in dry semen than in bulk semen. The goodness-of-fit for the linear regression represented as the r^2^ value is 0.53 for bulk semen and 0.82 for dry semen with an initial titer of 10^6^ TCID_50_/mL, respectively, and 0.65 for bulk semen with an initial titer of 10^3^ TCID_50_/mL. No curve is shown for the initial titer 10^3^ TCID_50_/mL in the dry semen because no viable virus was recovered after day 1. The titer on day 1 was 1.1 log_10_ TCID_50_/mL. In all cases except the high-titer bulk semen sample, the final data point was followed by 2 consecutive days of no recovered virus. B) C_t_ values produced by analysis of bulk semen samples analyzed by real-time quantitative reverse transcription PCR. The data did not fit a linear regression model (r^2^ = 0.08964), but the RNA clearly remained stable during the entire experiment. Three biologic replicates were analyzed at each time point. Error bars represent mean ± SEM virus titer. Dashed line indicates the limit of detection for the titration assay. An analysis of covariance was used to compare linear regression models and determine differences in virus reduction rates. C_t_, cycle threshold; TCID_50_, 50% tissue culture infectious dose.

We tested he stability of EBOV in dry and bulk semen for 8 days under tropical conditions (27°C, 80% RH). Under bulk conditions, EBOV was viable for all 8 days at 1 × 10^6^, but at 1 × 10^3^, viable virus was recovered only to day 6. EBOV viability was greatly reduced in dried semen: viable virus was detected to days 4 and 1 at 1 × 10^6^ and 1 × 10^3^, respectively (Figure 2). Viable virus was recovered from semen spiked with 1 × 10^3^ TCID_50_/mL EBOV stored in condoms to day 6, whereas the C_t_ value remained stable throughout the experiment. This finding highlights the importance of the proper disposal of condoms used by EVD convalescent men. EBOV RNA was detectable in semen for all 8 days with no decrease in C_t_ values, suggesting that RNA can be detected in semen samples obtained from convalescent men over an extended time, even if the cold chain is interrupted.

Before the EVD case in the woman reported here, EBOV was known to persist for an extended period in semen (*7*). In November 2014, a man returning to India from Liberia after recovery from EVD produced a positive semen sample while testing negative for EBOV in blood, saliva, and urine by qRT-PCR (*8*,*9*). Isolation of EBOV from semen samples collected during prior outbreaks has been reported. In 1 case, EBOV was isolated from the semen of a convalescent patient 82 days after symptom onset (*10*). EBOV has been detected at 101 days from symptom onset by qRT-PCR (*11*). Sexual transmission has been implicated in Marburg virus infection, but until now only equivocal evidence existed of EBOV transmission through sexual contact (*12*,*13*).

Our study has several limitations. First is our use of semen spiked with EBOV rather than naturally infected by EBOV. If EBOV is cell associated in a natural infection, the results of the experiment might be altered, although no difference was observed with blood previously (*4*). Second, the starting titers of EBOV in the semen might not represent naturally occurring levels of viable virus in semen because reported C_t_ values cannot be inferred toward viable virus or infectiousness. Third, the absolute C_t_ values presented directly apply to the instruments and reagents used in these experiments. Other systems might yield different C_t_ values and PCR efficiencies.

## Conclusions

Because of the potential for sexual transmission, the World Health Organization and Centers for Disease Control and Prevention have recommended measures to prevent transmission by sexual contact, including semen screening for survivors and use of condoms (*14*,*15*) and safe handling and disposal of condoms (*2*). In a region where organized waste management is almost nonexistent and availability of condoms is limited, to the extent that persons may wash and reuse condoms, this recommendation might not be strictly adhered to. The prolonged viability of EBOV in semen ex vivo supports the WHO recommendation for safe handling and disposal of condoms (*2*).
